# Sepsis Risk Factors in Neonatal Intensive Care Units of Public Hospitals in Southeast Ethiopia, 2020: A Retrospective Unmatched Case-Control Study

**DOI:** 10.1155/2023/3088642

**Published:** 2023-11-11

**Authors:** Gemechu Ganfure, Bikila Lencha

**Affiliations:** ^1^Department of Pediatrics and Child Health Nursing, Ambo University, Ambo, Ethiopia; ^2^Department of Public Health, School of Health Sciences, Madda Walabu University, Shashemene Campus, Shashemene, Ethiopia

## Abstract

**Background:**

Neonatal sepsis is a significant contributor to neonatal morbidity and mortality worldwide. It is more prevalent in developing countries. Thus, understanding the risk factors for neonatal sepsis is critical to minimizing the incidence of infection, particularly in Ethiopia. The purpose of this study was to identify the risk factors for neonatal sepsis in neonates admitted to neonatal intensive care units of public hospitals in Southeast Ethiopia in 2020.

**Method:**

An institution-based, retrospective unmatched case-control study was conducted on 97 cases and 194 controls in neonatal intensive care units of public hospitals in Southeast Ethiopia. A pretested, structured questionnaire was used to collect the data. Data was entered using EpiData 3.1 and analyzed using SPSS version 23. Bivariable and multivariable logistic regression analyses were performed to identify associated factors. An adjusted odds ratio with a 95% confidence interval was used to determine the degree of association, and statistical significance was declared at a *p* value of < 0.05.

**Results:**

In this study, 97 cases and 194 controls were included. About two-thirds (63.9%) of cases were with early onset neonatal sepsis (<7 days). Mode of delivery with spontaneous vaginal delivery (AOR:5.032; 95% CI (1.887-13.418)), type of birth attendant (traditional birth attendant) (AOR: 4.407 95% CI (1.213,16.004)), history of STI/UTI (AOR:2.543; 95% CI (1.313,4.925)), intrapartum fever (AOR:4.379; 95% CI (2.170,8.835)), APGAR score at the 5^th^minute < 7 (AOR:4.832; 95% CI (1.862,12.537)), neonate received resuscitation (AOR:3.830; 95% CI (1.753,8.369)), low birth weight (AOR:6.101; 95% CI (2.124,17.525)) were the identified risk factors for neonatal sepsis.

**Conclusion:**

Both maternal and neonatal factors contribute to the risk of neonatal sepsis. Spontaneous vaginal delivery, birth attended by the traditional birth attendant, history of STI/UTI, presence of intrapartum fever, low APGAR score at the 5^th^ minute, neonate receiving resuscitation, and low birth weight were identified as independent risk factors for neonatal sepsis. Prompt identification of the aforementioned factors and management should be sought for all newborns.

## 1. Introduction

Neonatal sepsis is a clinical condition of bacteremia with systemic signs and symptoms of infection in the first 28 days of life. It can also result from viral and fungal invasion of the bloodstream [1]. Neonatal sepsis can occur in the first week of the neonatal period, early-onset sepsis (EOS), or after the first week of the neonatal period, late-onset sepsis (LOS) [2].

Globally, 2.4 million children died in the first month of life in 2020. Approximately 6,500 neonatal deaths occur every day, with about a third of all neonatal deaths occurring within the first day after birth, and close to three-quarters occurring within the first week of life [3]. The Global Burden of Disease (GBD) estimates 1.3 million annual incident cases of neonatal sepsis [4].

The burden of neonatal sepsis is high among neonates in low- and middle-income countries (LMICs), and there is poor access to health care facilities and trained health professionals [5–7]. About 9.8% of the case fatality risk of neonatal sepsis in the first month of life occurs in sub-Saharan Africa, Latin America, and South Asia and causes major infirmity for 39% of those affected, even with timely antibiotic treatment [8, 9].

In developing countries, neonatal sepsis contributes to 30–50% of neonatal deaths [10]. Similarly, approximately 89,000 neonates die in the first four weeks of life in Ethiopia, accounting for 44% of all deaths among children under the age of five [11]. The reported neonatal mortality rate (NMR) in the 2019 mini Ethiopian demographic health survey (EDHS) was 30 deaths per 1,000 live births, which was nearly identical to the figure in the 2016 EDHS report, which was 29 deaths per 1,000 live births [12]. Neonatal sepsis was found to be the major cause of neonatal death in Ethiopia [13, 14].

Premature rupture of membranes (PROM), maternal health conditions [15–17], history of maternal urinary tract infection or sexually transmitted infection, presence of intrapartum fever, gestational age of 37 weeks, low APGAR score of 7 at the 5th minute, need for resuscitation, not crying immediately at birth [18, 19], and lack of access to skilled birth attendants, mechanical ventilation, prelacteal feeding, and intravenous cannula [20–23] were identified as contributing factors of neonatal mortality in previous studies.

Identification of the prevalence, etiology, risk factors, and outcome of the problem aids in early detection and management [24]; delay in commencing treatment increases the mortality rate from neonatal sepsis by 50% [25]. To avoid this problem, understanding the common risk factors for neonatal sepsis in a given area becomes critical for guiding local empirical antibiotic selection and preventing drug resistance, especially in the current study area [4].

## 2. Objectives

This study was aimed at identifying factors associated with neonatal sepsis in neonatal intensive care units of public hospitals in Southeast Ethiopia.

## 3. Methods

### 3.1. Study Area and Design

An institution-based, retrospective unmatched case-control study was conducted at a public hospital in Southeast Ethiopia from January 1^st^ to April 30^th^, 2020. All neonates who were admitted to the neonatal intensive care unit (NICU) were the source population. Similarly, all neonates admitted to the NICU during the study period were the study population.

### 3.2. Study Population

In this study, all neonates admitted to the neonatal intensive care unit with their index mothers were the study population of the study. Cases were neonates diagnosed for neonatal sepsis, whereas controls were neonates admitted with indications other than neonatal sepsis to the neonatal intensive care units who fulfill the inclusion criteria.

### 3.3. Inclusion and Exclusion Criteria for Cases and Controls

#### 3.3.1. Inclusion Criteria for Cases and Controls

This study included cases of neonates diagnosed with neonatal sepsis using established clinical and hematological criteria of IMNCI with their mother. Controls were neonates who were admitted to the hospital for reasons other than neonatal sepsis and were not suspected or diagnosed with the sepsis.

#### 3.3.2. Exclusion Criteria for Cases and Controls

Neonates clinically suspected for sepsis, abandoned neonates, mothers in critical illness, and neonates' charts with incomplete information were excluded from the study.

### 3.4. Operational Definition

#### 3.4.1. Case

All neonates admitted to the neonatal intensive care unit fulfilled the diagnostic criteria for neonatal sepsis.

#### 3.4.2. Neonatal Sepsis Diagnostic Criteria

The established integrated management of neonatal and childhood illness (IMNCI) clinical features including the presence of two or more of persistent fevers (≥37.5°C) or persistent hypothermia (≤35.5°C) for more than one hour, fast breathing (≥60 breaths per minute), severe chest indrawing, grunting, not feeding well, movement only when stimulated, bulged fontanel, convulsion, lethargic or unconsciousness along with ≥2 of the hematological criteria such as total leukocyte count (<4000 or>12,000 cells/mm^3^), absolute neutrophil count (<1500 cells/mm^3^ or >7500 cells/mm^3^), platelet count (<150 or>450 cells/mm^3^), and random blood sugar (<40 mg/dl or>125 mg/dl) were used to diagnose neonatal sepsis cases.

#### 3.4.3. Control

Neonates with the diagnosis of nonsepsis cases with their index mother and fulfill the inclusion criteria.

### 3.5. Sample Size Determination and Sampling Procedure

The sample size was estimated considering two population proportion formulas by using OpenEpi version 3.01. The proportion of mothers with UTI/STI (13% (main exposure variable)) from the study conducted in Ghana and the 90% power of the study control to case ratio of 2 : 1 to detect an odds ratio of 3.007 were used to determine the sample size [15]. The calculated sample size was 88 cases and 176 controls, giving a total sample size of 264. In this study, 97 cases and 194 controls yielded a total sample size of 291 after accounting for a 10% nonresponse rate. Cases and controls were selected using consecutive sampling techniques from neonates admitted to the NICU until the required sample size was met. In addition, information was obtained from the respective mothers, and charts were reviewed for both case and control.

### 3.6. Data Collection Methods and Procedures

The structured questionnaire prepared in English was adopted from a previous study to collect data [18]. The questionnaire contains sociodemographic data, maternal and neonatal risk factors for neonatal sepsis, and the IMNCI checklist for neonatal sepsis diagnosis and hematologic laboratory findings. All eligible neonates fulfilling inclusion criteria for cases and controls admitted to NICU were selected from neonatal admission registration. Neonates' medical charts were checked for completeness and clarity to be included in the study. Checklist was used to collect information about neonatal factors of sepsis. The interview was used to collect data on maternal factors of neonatal sepsis from index mothers.

### 3.7. Data Quality Control

The checklist was pretested on 5% of the sample size before the actual data collection to confirm the reliability of the data. The data collection was performed by two BSC nurses who have data collection experience after receiving training on the objectives, study participant selection, contents, accuracy, and consistency of the data for two days. The data were checked for completeness during the data collection by site supervisors and investigators. Double data entry was used to ensure the quality of the data.

### 3.8. Data Management and Analysis

Data was entered using EpiData 3.1 and exported to SPSS version 23 for analysis. Descriptive statistics were used to assess the sociodemographic characteristics of the mother and neonates, and the result was summarized as frequencies and percentages. Binary and multiple logistic regressions were used, to determine factors associated with neonatal sepsis. Variables with a *p* value of < 0.25 in bivariate analyses were included in the multiple logistic models. *p* values less than 0.05 were considered sufficient to declare statistical significance in the model. The result was reported using crude and adjusted odds ratios (OR) with their 95% confidence interval.

## 4. Results

### 4.1. Sociodemographic Characteristics of Mothers and Neonates

A total of 97 cases and 194 controls were included with their index mother in the study. The mean age of the mothers' age was 25.78 ± 4.332. Fifty-four (26.9%) of cases' mothers and 120 (61.9%) of controls' mothers were from urban areas. Regarding the marital status of the mothers, 95.9% of the cases' and 99% of the controls' mothers were married. In the majority, 72.2% of cases' mothers and 52.6% of controls' mothers were housewives, and 36.1% of cases' mothers and 29.9% of controls had attended primary education. About neonates' sociodemographic characteristics, 63.9% of the cases and 90.2% of the controls were found to be below the age of 7 days. Fifty-eight (59.8%) of the cases and 105 (54.1%) of the controls were male neonates (Table 1).

### 4.2. Maternal-Related Factors of Neonatal Sepsis

For both cases and controls, the median (IQ range) parity was 2, with a range of 1 to 8 live births. During the pregnancy period of the current neonate, 190 (97.9%) of the mothers of controls and 92 (94.8%) of the mothers of the case had at least one ANC visit. More than half, 59 (60.8%) of the cases and almost all 175 (90.2%) controls were delivered at the hospital, and 114 (58.8%) of the controls and 70 (72.2%) of the cases were delivered by spontaneous vaginal delivery. One hundred eighty-six (95.9%) of the controls and 84 (86.6%) of the cases were attended by skilled birth attendants like doctors, nurses, and midwives. This study showed that the proportion of the history of sexually transmitted infections or urinary tract infections (STI/UTI) during the current pregnancy is higher in cases 55(56.7%) than in controls, 52(26.8%) (Table 2).

### 4.3. Clinical Signs of Neonatal Sepsis

Seventy (72.2%) of the cases and 38 (19.6%) of controls had fast breathing with a breath-per-minute rate greater than 60, and about 12 (12.4%) of the cases and 8 (4.1%) of controls had resuscitation at birth. Grunting was present in 59 (60.8%) of the cases and 7 (3.6%) of controls, and high or low temperatures were present in 84 (84.6%) of cases and 85 (43.8%). Poor feeding and severe chest indrawing were reported in cases 36 (37.1%) and 34 (35.1%) and in controls 4 (2.1%) and 3 (1.5%), respectively (Table 3).

### 4.4. Neonate-Related Factors of Neonatal Sepsis

Nearly all 156 (80.4) of the controls and 42 (43.3) of the cases were born with normal birth weight. One hundred thirty-four (69.1%) controls and 56 (57.7%) cases were delivered at term. About 27 (27.8%) of cases and 12 (6.2%) controls had APGAR (appearance, pulse rate, grimace, activity, and respiration) scores of less than seven (≥) at the fifth minute. Similarly, 43 (44.3%) of cases and 20 (10.3%) of controls had received resuscitation at birth (Table 4).

### 4.5. Maternal and Neonatal Risk Factors for Neonatal Sepsis

Both bivariable and multivariable binary logistic regressions were applied. In bivariate binary logistic regression, significance was seen in maternal education, occupation of the mother, neonatal age, frequency of ANC visit, duration of labor, mode of delivery, type of birth attendant, antepartum hemorrhage, history of STIs/UTIs, intrapartum fever, low APGAR score at the 5^th^ minute, neonate receiving resuscitation, and low birth weight.

In multivariable logistic regression, mode of delivery, type of birth attendant, history of STI/UTI, presence of intrapartum fever, APGAR score at the 5^th^ minute, neonate receiving resuscitation, and low birth weight were associated with neonatal sepsis.

In the current study, mode of delivery showed a significant association with neonatal sepsis. Neonates who had delivered with spontaneous vaginal delivery had 5 times odds of developing sepsis when compared to the neonates delivered with cesarian section (AOR:5.032; 95% CI (1.887-13.418)). The type of birth attendant showed significant association with the risk of neonatal sepsis. Neonates attended by traditional birth attendants had 4 times odds of having risk for developing neonatal sepsis (AOR: 4.407 95% CI (1.213,16.004)). Likewise, history of the sexually transmitted infection or urinary tract infection during pregnancy was found to have significant association with neonatal sepsis. Neonates born to mothers who had STI/UTI during pregnancy had 2.5 times higher odds of developing sepsis than neonates born to mothers who did not have STI/UTI (AOR:2.543; 95% CI (1.313,4.925)). Intrapartum fever was found to be a risk factor for the neonatal sepsis. In this study, neonates born to mothers having intrapartum fever had 4 times higher odds of developing sepsis when compared to the neonates born to their counterparts (AOR:4.379; 95% CI (2.170,8.835)).

Similarly, APGAR score at the fifth minute was significantly associated with the risk of neonatal sepsis. Neonates who had APGAR of <7 at the fifth minute had 4.8 times higher odds of having sepsis when compared to the neonates who had APGAR score of ≥7 (AOR:4.832; 95% CI (1.862,12.537)). In this study, neonatal resuscitation showed a significant association with the occurrence of neonatal sepsis. Neonates who had received resuscitation had 3.8 times higher odds of developing sepsis when compared to neonates who had not received resuscitation (AOR:3.830; 95% CI (1.753,8.369)). Also, significant association had been seen between birth weight and neonatal sepsis. Neonates with low birth weight < 2500 mg had 6 times higher odds of having sepsis when compared to neonates with normal birth weight ≥ 2500 mg (AOR:6.101; 95% CI (2.124,17.525)) (Table 5).

## 5. Discussion

This study was aimed at assessing risk factors for neonatal sepsis and generating information that helps as an input in the early detection and management of risk factors for neonatal sepsis.

Maternal education, occupation of the mother, neonatal age, frequency of ANC visit, duration of labor, mode of delivery, type birth attendant, antepartum hemorrhage, history of STIs/UTIs, intrapartum fever, low APGAR score at the 5^th^ minute, neonate receiving resuscitation, and low birth weight were maternal and neonatal factors for neonatal sepsis included in the study. Of these factors, mode of delivery, type of birth attendant, history of STI/UTI, intrapartum fever, APGAR score at the 5^th^ minute, neonate receiving resuscitation, and low birth weight were found to have a significant association with neonatal sepsis.

About two-thirds (63.9%) of cases were with early onset neonatal sepsis (<7 days), which is almost comparable with the studies conducted in Hawassa [26], Mekele [18] Bishoftu [27], and Gondar [28]. This might be explained by the fact that the majority of neonatal sepsis cases occur during the first week of neonatal life.

The present study indicated that mode of delivery had a significant association with neonatal sepsis. Neonates who were born with spontaneous vaginal delivery had 5 times higher odds of developing sepsis when compared to neonates born with cesarean section. Unlike the current study, another study did not show the association between neonatal sepsis and mode of delivery [18, 23, 26]. The probable reason for the association could be that during spontaneous vaginal delivery, there might be repeated per vaginal examination in which microorganisms introduce into the birth canal and ascended into the upper genital tract in which the fetus might be exposed to before delivery.

Birth attendant showed significant association with neonatal sepsis. Neonates attended by traditional birth attendants had 4.4 times higher odds of having neonatal sepsis when compared to neonates attended by health professionals. The association is most likely due to the fact that neonates delivered at home while being attended by traditional birth attendants may not have received the proper essential newborn care. The other reason might be that a neonate would not receive proper cord care, which would expose the neonate to contaminated cord care items like blades and cord tie.

Association was seen between the presence of intrapartum fever and neonatal sepsis. Neonates born to mothers having intrapartum fever had 4.4 times higher risk of developing sepsis when compared to neonates born to mothers who did not experience fever during labor. Intrapartum fever was one of the risk factors contributing to the development of neonatal sepsis. The result was in line with the findings of other studies in which intrapartum fever was found to be the risk factor for neonatal sepsis [2, 18, 26]. Early and late-onset neonatal sepsis commonly occurs later from mothers having history of fever during the childbirth which may indicate the mother probably had infections.

In the current study, history of sexually transmitted infection/urinary tract infection during pregnancy was found to be a risk factor for neonatal sepsis. A neonate born to a mother with history of STI/UTI during pregnancy had 2.5 times higher odds of developing sepsis than neonates born to a mother who had no history of STI/UTI during the index pregnancy. This finding was consistent with the study done elsewhere in which history of STI/UTI was found to be the risk factor for neonatal sepsis [18, 27, 29]. This could be due to the exposure of the fetus and infant to the maternal organism in utero and during the passage through the birth canal, respectively. The other probable reason might be premature and low birth weight neonates are more at risk of developing neonatal sepsis when exposed to maternal microorganism.

This study revealed that APGAR scores less than seven at the fifth minute showed an association with neonatal sepsis. Neonates who had APGAR scores of <7 at the 5^th^ minute had four times the odds of developing sepsis when compared to neonates who had APGAR scores of ≥7. A similar study showed a significant association between the APGAR score at the 5^th^ minute and neonatal sepsis [15, 17, 24]. The respiratory response in the first minute is crucial for the survival and well-being of the newborn. Birth asphyxia, which predisposes the newborn for resuscitation, is a risk factor for sepsis. Thus, a low APGAR score is mostly associated with neonatal sepsis [30–32]. A neonate with low APGAR score might have the high probability of receiving resuscitation in which the chance of being exposed to microorganism from resuscitation instrument and health professionals.

Neonatal birth weight showed a significant association with neonatal sepsis. In this study, a neonate with a birth weight of less than 2500 mg (LBW) showed a higher risk of acquiring neonatal sepsis than neonates having a birth weight greater than 2500 gm. This is comparable with other studies in which low birth weight was found to be a risk factor for neonatal sepsis [28, 33–35]. The ability of low-birth-weight neonates to defend against infections is poor, and they are more likely to develop an infection. Mostly parenteral nutrition happens in low-birth-weight neonates, and there is the risk of infection from contamination of IV cannulas [36].

The presence of neonatal resuscitation was seen as a risk factor for neonatal sepsis. Neonates who received resuscitation after birth had 3.8 times higher odds of having sepsis when compared to neonates who did not receive resuscitation. This finding was similar with the study done elsewhere in which neonatal resuscitation was found to be a risk factor for neonatal sepsis [17, 37–40]. The probable explanation for this association might be that neonates who received resuscitation may have been exposed to potential sources of microorganisms from resuscitation equipment and cross-contamination by health professionals [41–43].

## 6. Conclusion

Both maternal and neonatal factors contribute to the risk of neonatal sepsis. Spontaneous vaginal delivery, birth attended by traditional birth attendant, history of STI/UTI, presence of intrapartum fever, low APGAR score at the 5^th^ minute, neonate receiving resuscitation, and low birth weight were identified as independent risk factors for neonatal sepsis. Neonates with low APGAR scores at fifth minute of life should be closely monitored, as they may receive resuscitation and become infected as a result of the procedure. Neonates with low birth weight should be managed accordingly to minimize cross-contamination during handling and feeding. Early antenatal screening, detection and management of problems, proper intrapartum care and prevention of infection, and timely management of complications should be sought for all newborns.

## 7. Limitation of the Study

The current study has a limitation of not confirming a causal relationship and recall bias.

## Figures and Tables

**Table 1:** Sociodemographic characteristics of mothers and neonate at public hospitals in Southeast Ethiopia, 2020.

Variables		Case *n* (97)		Control *n* (194)	
		Frequency	Percent	Frequency	Percent
Maternal age	<18	5	5.2	6	3.1
	18-25	52	53.6	79	40.7
	>25	40	41.2	109	56.2

Marital status	Married	93	95.9	192	99.0
	Others	4	4.1	2	1.0

Religion	Orthodox	22	22.7	62	32.0
	Muslim	66	68.0	104	53.6
	Others	9	9.3	28	14.4

Ethnicity	Oromo	85	87.6	172	88.7
	Others	12	12.4	22	11.3

Residence	Urban	54	55.7	120	61.9
	Rural	43	44.3	74	38.1

Maternal education	No education	32	33.0	52	26.8
	Primary	35	36.1	58	29.9
	Secondary	15	15.5	33	17.0
	College and higher	15	15.5	51	26.3

Occupation of mother	Housewife	70	72.2	102	52.6
	Government employee	15	15.5	48	24.7
	Others	12	12.3	44	22.7

Neonatal sex	Male	58	59.8	105	54.1
	Female	39	40.2	89	45.9

Neonatal age	<7 days	50	51.5	71	36.6
	≥7 days	47	48.5	123	63.4

**Table 2:** Maternal factors of neonatal sepsis at public hospitals in Southeast Ethiopia, 2020.

Variables		Case *n* (97)		Control *n* (194)	
		Frequency	Percent	Frequency	Percent
ANC visit	Yes	92	94.8	190	97.9
	No	5	5.2	4	2.1

Parity	Primipara	59	60.9	135	69.6
	Multipara	38	39.1	52	30.4

Frequency of ANC visit	<4	73	79.3	134	70.5
	≥4	19	20.7	56	29.5

Place of delivery	Home	13	13.4	5	2.6
	Hospital	59	60.8	175	90.2
	Health center	25	25.8	14	7.2

Duration of labor	≥12 hour	41	42.3	60	30.9
	<12 hour	56	57.7	134	69.1

Mode of delivery	SVD	70	72.2	114	58.8
	Instrumental delivery	11	11.3	39	20.1
	C/S	16	16.5	41	21.1

Birth attendant	TBA	13	13.4	8	4.1
	Health professional	84	86.6	186	95.9

Presence of intrapartum fever	Yes	66	68	52	26.8
	No	31	32	142	73.2

Foul-smelling amniotic fluid	Yes	19	19.6	28	14.4
	No	78	80.4	166	85.6

Pregnancy-related hypertension	Yes	6	6.2	12	6.2
	No	91	93.8	182	93.8

Antepartum hemorrhage	Yes	8	8.2	3	1.5
	No	89	91.8	191	98.5

History of STIs/UTIs	Yes	55	56.7	52	26.8
	No	42	43.3	142	73.2

**Table 3:** Clinical signs of neonatal sepsis of neonates at public hospitals in Southeast Ethiopia, 2020. Case = 97, Control = 194, *n* = 291.

Variables	Category	Case/control status	
		Case (%)	Control (%)
Resuscitation at birth	Yes	12 (12.4)	8 (4.1%)
	No	85 (87.6)	186 (95.9%)

Respiratory rate > 60 breaths/min	Yes	70 (72.2)	38 (19.6%)
	No	27 (27.8%)	156 (80.4%)

Grunting	Yes	59 (60.8%)	7 (3.6%)
	No	38 (39.2%)	187 (96.4)

Redness around umbilicus	Yes	11 (11.3%)	6 (3.1%)
	No	86 (88.7%)	188 (96.6%)

High/low temperature	Yes	84 (86.6%)	85 (43.8%)
	No	13 (13.4%)	109 (56.2%)

Lethargic/unconscious	Yes	15 (15.5%)	5 (2.6%)
	No	82 (84.5%)	189 (97.4%)

Poor feeding	Yes	36 (37.1%)	4 (2.1%)
	No	61 (62.9%)	190 (97.9%)

Severe chest indrawing	Yes	34 (35.1%)	3 (1.5%)
	No	63 (64.9%)	191 (98.5%)

**Table 4:** Neonatal factors for neonatal sepsis at public hospitals in Southeast Ethiopia, 2020.

Variables		Case *n* (97)		Control *n* (194)	
		Frequency	Percent	Frequency	Percent
Gestational age	Term	41	42.3	60	30.9
	Preterm	56	57.7	134	69.1

Birth weight	<2500	55	56.7	38	19.6
	≥2500	42	43.3	156	80.4

APGAR score 1^st^ minute	<7	77	79.4	116	59.8
	≥7	20	20.6	78	40.2

APGAR score 5^th^ minute	<7	27	27.8	12	6.2
	≥7	70	72.2	182	93.8

Neonate cry after birth	Yes	86	88.7	182	93.8
	No	11	11.3	12	6.2

Neonate resuscitated	Yes	43	44.3	20	10.3
	No	54	55.7	174	89.7

**Table 5:** Association of maternal and neonatal risk factors of neonatal sepsis public hospitals in Southeast Ethiopia, 2020.

Variables		Case *n* (97)		Control *n* (194)		COR (95% CI)	AOR (95% CI)
		Frequency	Percent	Frequency	Percent		
Maternal education	No education	32	33.0	52	26.8	2.092 (1.367, 3.202)^∗^	1.107 (.422, 2.905)
	Primary	35	36.1	58	29.9	2.052 (1.351, 1.80)^∗^	1.282 (.500, 3.291)
	Secondary	15	15.5	33	17.0	1.545 (.944, 2.529)	1.454 (.490, 4.311)
	College and higher	15	15.5	51	26.3	1.00	1.00

Occupation of mother	Housewife	70	72.2	102	52.6	2.516 (1.662, 3.811)^∗^	3.063 (.897, 10.458)
	Government employee	15	15.5	48	24.7	1.146 (.691, 1.901)	.935 (.179, 4.881)
	Others^∗^	12	12.3	44	22.7	1.00	1.00

Neonatal age	<7 days	62	63.9	175	90.2	1.843 (1.379, 2.463)^∗^	1.369 (0.706, 2.656)
	≥7 days	35	36.1	19	9.8	1.00	1.00

ANC visit	<4 visits	73	79.3	134	70.5	1.418 (1.017, 1.978)^∗^	2.112 (.943, 4.733)
	≥4visits	19	20.7	56	29.5	1.00	1.00

Labor duration	≥12 hours	41	42.3	60	30.9	1.635 (1.216, 2.199)^∗^	1.590 (.737, 3.429)
	<12 hours	56	57.7	134	39.1	1.00	1.00

Mode of delivery	SVD	70	72.2	114	58.8	1.573 (1.074, 2.304)^∗^	5.032 (1.887-13.418)^∗∗^
	Instrumental delivery	11	11.3	39	20.1	.723 (.430, 1.214)	1.587 (.496, 5.082)
	C/S	16	16.5	41	21.1	1.00	1.00

Birth attendant	TBA	13	13.4	6	3.1	4.849 (2.695, 8.726)^∗^	4.407 (1.213, 16.004)^∗∗^
	Health professional	84	86.6	188	96.1	1.00	1.00

Antepartum hemorrhage	Yes	8	8.2	3	1.5	5.723 (2.590, 12.643)^∗^	1.589 (.364, 6.943)
	No	89	91.8	191	98.5	1.00	1.00

STIs/UTIs	Yes	55	56.7	52	26.8	2.672 (1.019, 7.007)^∗^	2.543 (1.313, 4.925)^∗∗^
	No	42	43.3	142	73.2	1.00	1.00

Intrapartum fever	Yes	66	68.0	52	26.8	5.814 (4.255, 7.945)^∗^	4.379 (2.170, 8.835)^∗∗^
	No	31	32.0	142	73.2		1.00

APGAR score 5^th^ minute	<7	27	27.8	12	6.2	5.850 (3.803, 8.999)^∗^	4.832 (1.862, 12.537)^∗∗^
	≥7	70	72.2	182	93.8	1.00	1.00

Neonate resuscitated	Yes	43	44.3	20	10.3	6.928 (4.837, 9.922)^∗^	3.830 (1.753, 8.369)^∗∗^
	No	54	55.7	174	89.7	1.00	1.00

Birth weight	<2500	35	36.1	7	3.6	5.376 (3.925, 7.362)^∗^	6.101 (2.124, 17.525)^∗∗^
	≥2500	62	63.9	187	96.4	1.00	1.00

Others: private, daily laborers, and students. ^∗^Bivariate; ^∗∗^multivariate. SVD: spontaneous vaginal delivery; C/S: cesarean section; UTI/STIs: urinary tract infection/sexually transmitted infections.

## Data Availability

The datasets used and/or analyzed during the current study are available from the corresponding author on reasonable request.

## Abbreviations

AOR: Adjusted odds ratio
EDHS: Ethiopia demographic health survey
EONS: Early onset neonatal sepsis
GOHD: Global Health Observatory data
LONS: Late-onset neonatal sepsis
MDG: Millennium development goal
NICU: Neonatal intensive care unit
NS: Neonatal sepsis
PROM: Prolonged rupture of membrane
SDG: Sustainable development goal
STI: Sexually transmitted infection
UNIGCME: United Nations Inter-agency Group for Child Mortality Estimation Report
UTI: Urinary tract infection
WHO: World Health Organization.
